# Book Review

**Published:** 2009-03

## Book review

**Figure:**
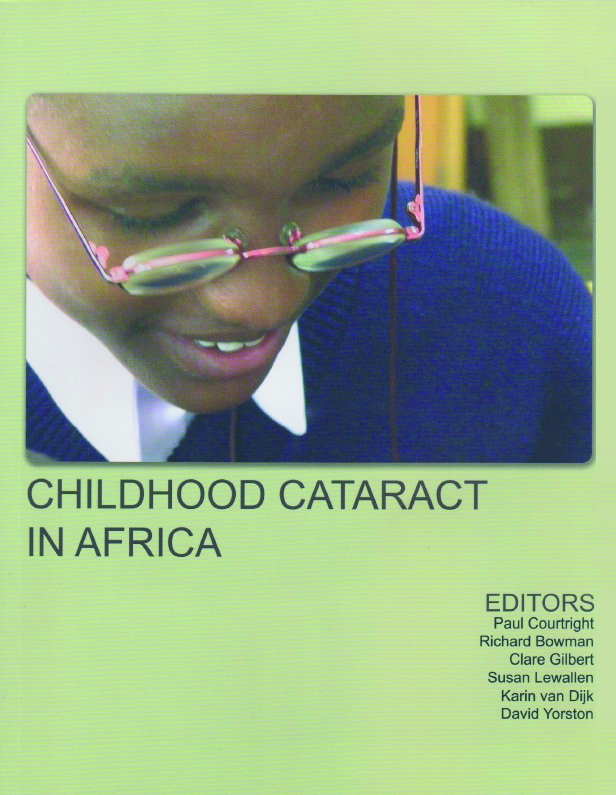


**Childhood cataract in Africa. Courtright P, Bowman R, Gilbert C, Lewallen S, van Dijk K, and Yorston D (editors).** 2008. Reviewed by J Nick Astbury, Consultant Ophthalmic Surgeon, Norfolk and Norwich University Hospital NHS Trust, Colney Lane, Norwich NR4 7UY UK.

This is quite simply a brilliant manual, beautifully illustrated and written by people immersed in the ethos of community eye health and with first-hand experience of dealing with childhood cataract in Africa. It incorporates the conclusions and recommendations from a meeting of experts that took place at the Kilimanjaro Christian Medical Centre (KCMC) in Moshi, Tanzania and it covers planning and strategies as well as pre- and postoperative management, counselling, and rehabilitation. Dark & Light Blind Care (Netherlands) must be congratulated for sponsoring this work which should be read by everybody involved with childhood cataract, including parents, health workers, and ophthalmologists. To order this book, go to **www.kcco.net** or contact: Teaching Aids at Low Cost (TALC), PO Box 49, St Albans, Herts, AL1 5TX, UK. Tel: +44 1727 853869

Email: info@talcuk.org

Website: www.talcuk.org

